# Supplementary material for: Increased SEC23A Expression Correlates with Poor Prognosis and Immune Infiltration in Stomach Adenocarcinoma
Source: Cancers (Basel). 2023 Mar 30;15(7):2065. doi: 10.3390/cancers15072065 (PMC10093042; doi:10.3390/cancers15072065)
Supplement: Supplementary file 1 [file cancers-15-02065-s001.zip › cancers-2269026-figure S1.pdf]

Figure S1. Flow chart for the bioinformatic expression analyses of *SEC23A:* clinicopathological features and survival

SEC23A expression was analyzed in 33 cancer entities, including 9807 tumor and 727 paratumor samples from TCGA and 7568 normal tissue samples from GTEx (Fig. 1A).

SEC23A levels of 132 samples included 111 STAD and 21 paratumor samples (Fig. 1C).

SEC23A levels of 30 samples included 15 STAD and 15 paratumor samples (Fig. 1D).

GTEx

TCGA

GSE54129

GSE118916

Transcript levels of *SEC23A* in 624 STAD cases, including 414 tumor and 36 paratumor samples from TCGA, as well as 174 normal gastric tissue samples from GTEx (Fig. 1B).

370 STAD patients with valid prognostic information and *SEC23A* expression values for tumors were included for the prognosis analysis (Fig. 3 A–C).

407 STAD patients with valid clinicopathological features together with *SEC23A* expression data were included for statistical analysis of relationships (Fig. 2).

347 STAD patients with complete clinicopathological features, prognostic information and *SEC23A* expression data were included in the COX analysis (Fig. 3D and Table 1).
