# Supplementary figures and images for: Increased SEC23A Expression Correlates with Poor Prognosis and Immune Infiltration in Stomach Adenocarcinoma
Source: Cancers (Basel). 2023 Mar 30;15(7):2065. doi: 10.3390/cancers15072065 (PMC10093042; doi:10.3390/cancers15072065)

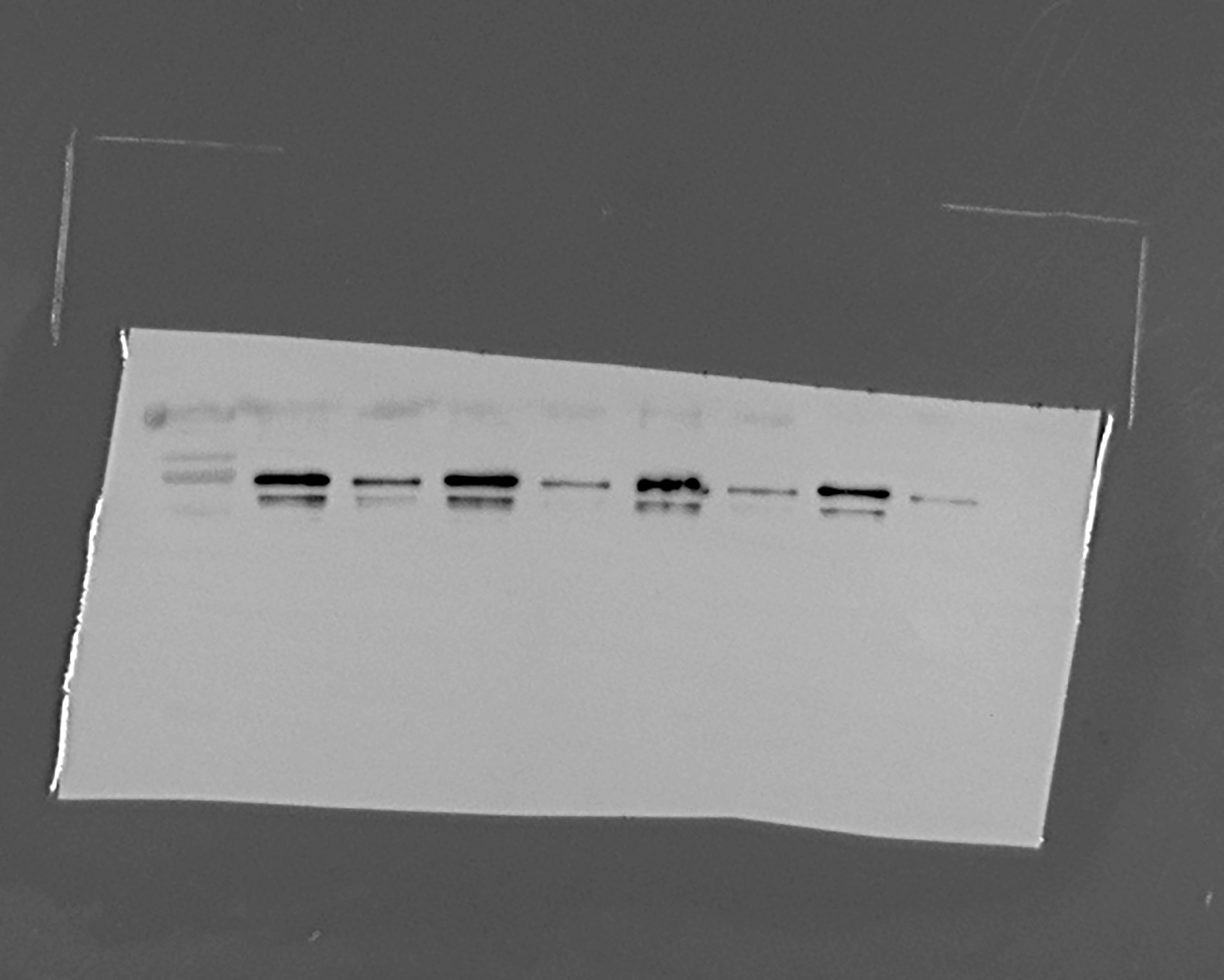

Supplement: Supplementary file 1 [file cancers-15-02065-s001.zip › File S1/SEC23A/SEC23A-1.tif]

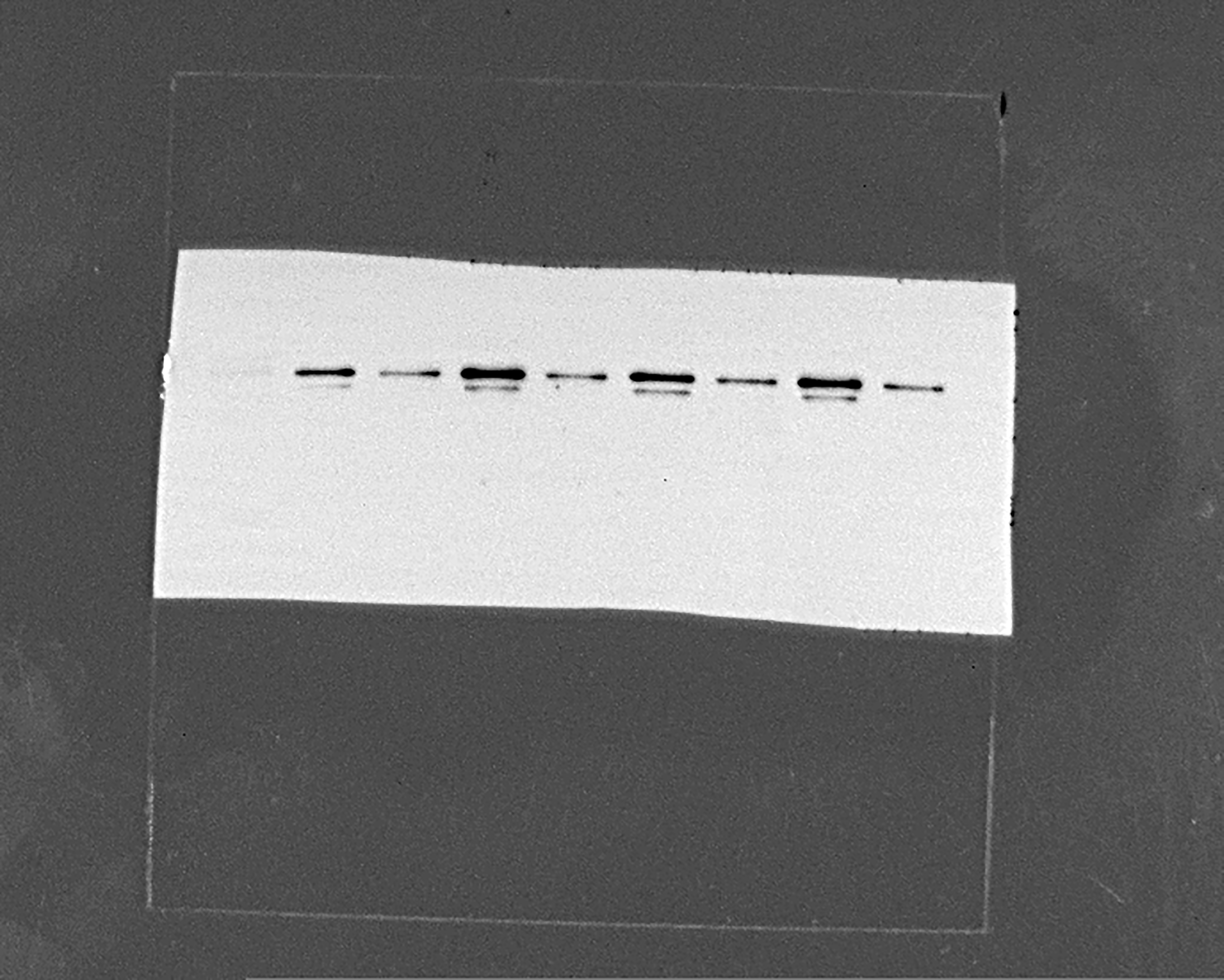

Supplement: Supplementary file 1 [file cancers-15-02065-s001.zip › File S1/SEC23A/SEC23A-2.tif]

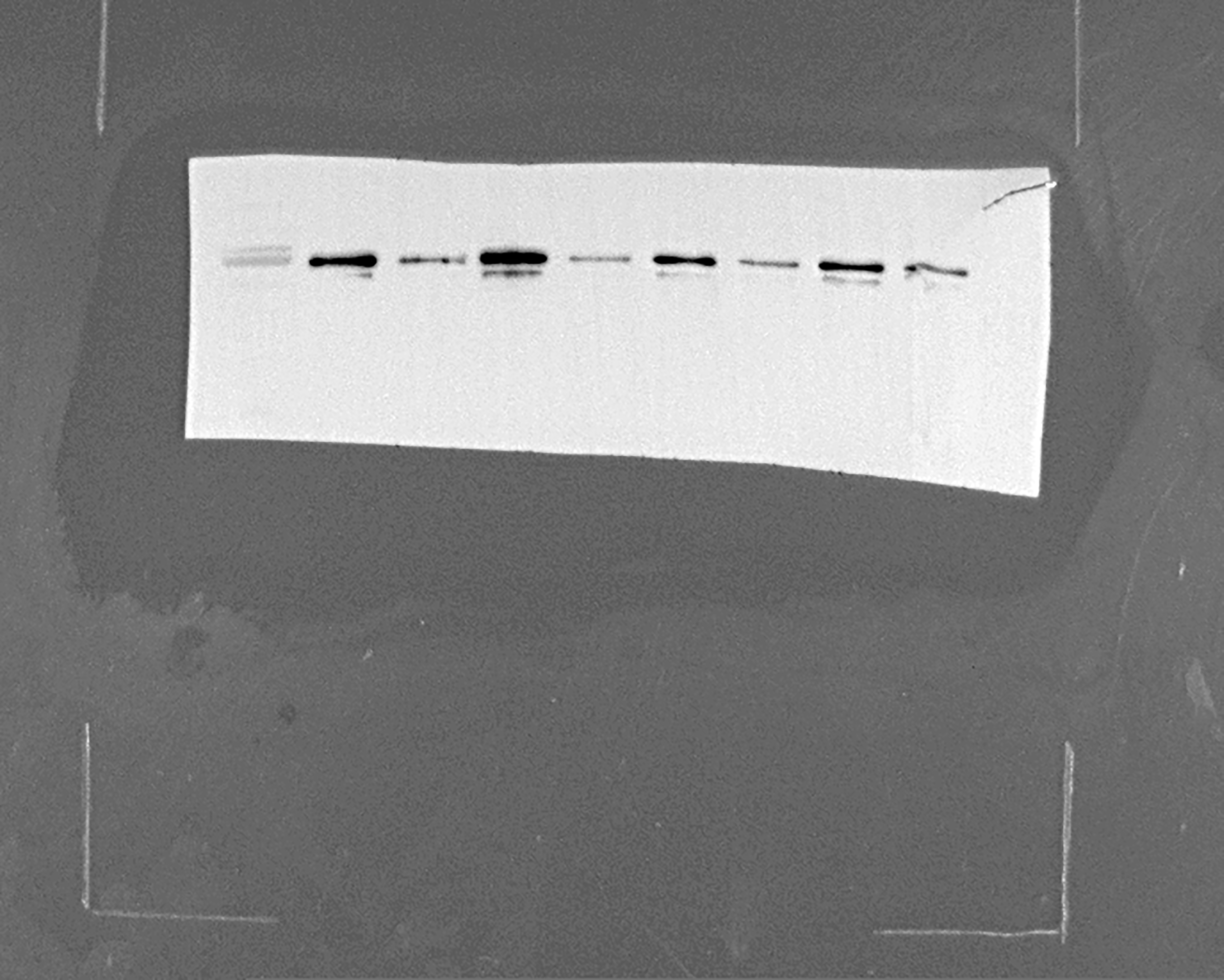

Supplement: Supplementary file 1 [file cancers-15-02065-s001.zip › File S1/SEC23A/SEC23A-3.tif]

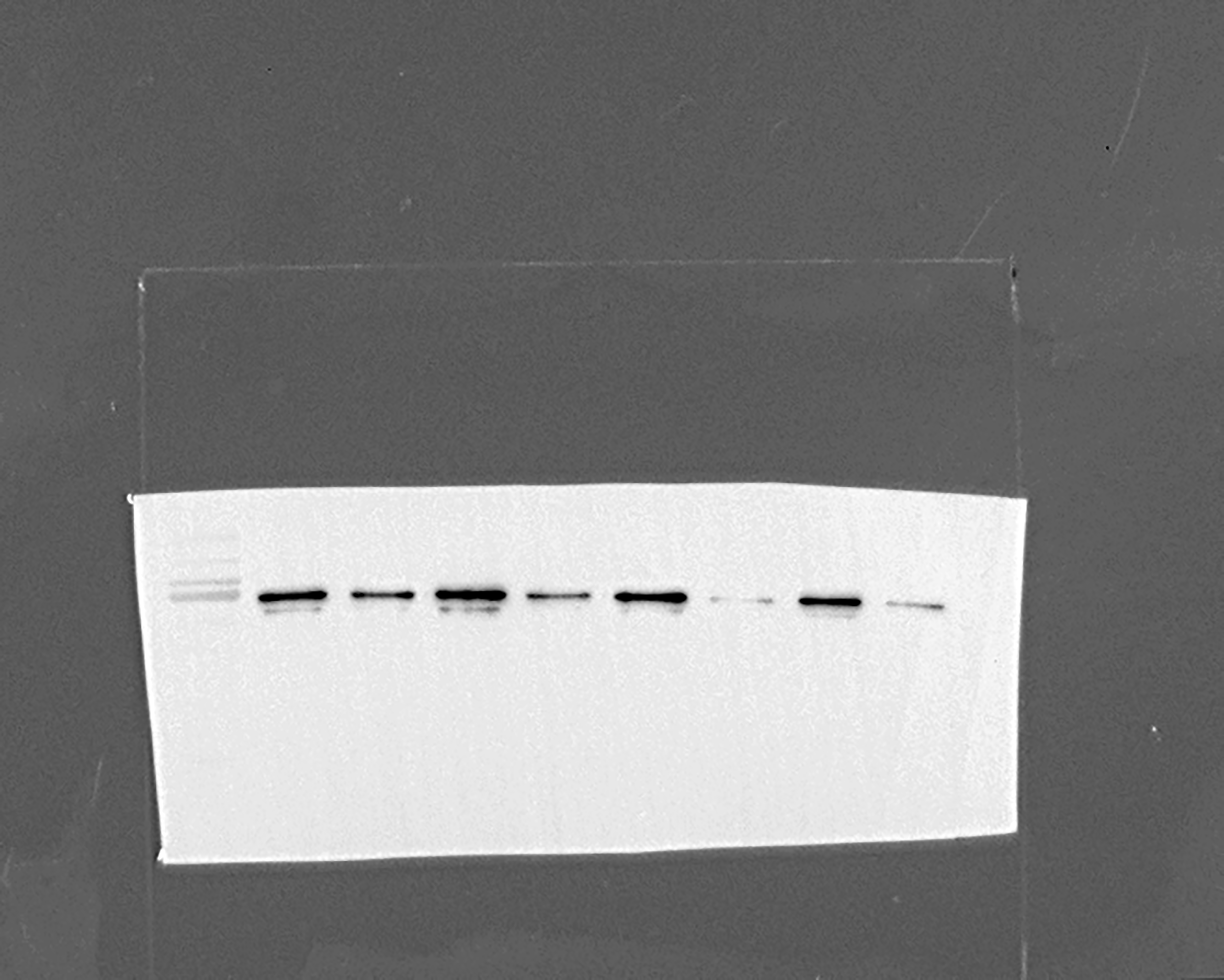

Supplement: Supplementary file 1 [file cancers-15-02065-s001.zip › File S1/SEC23A/SEC23A-4.tif]

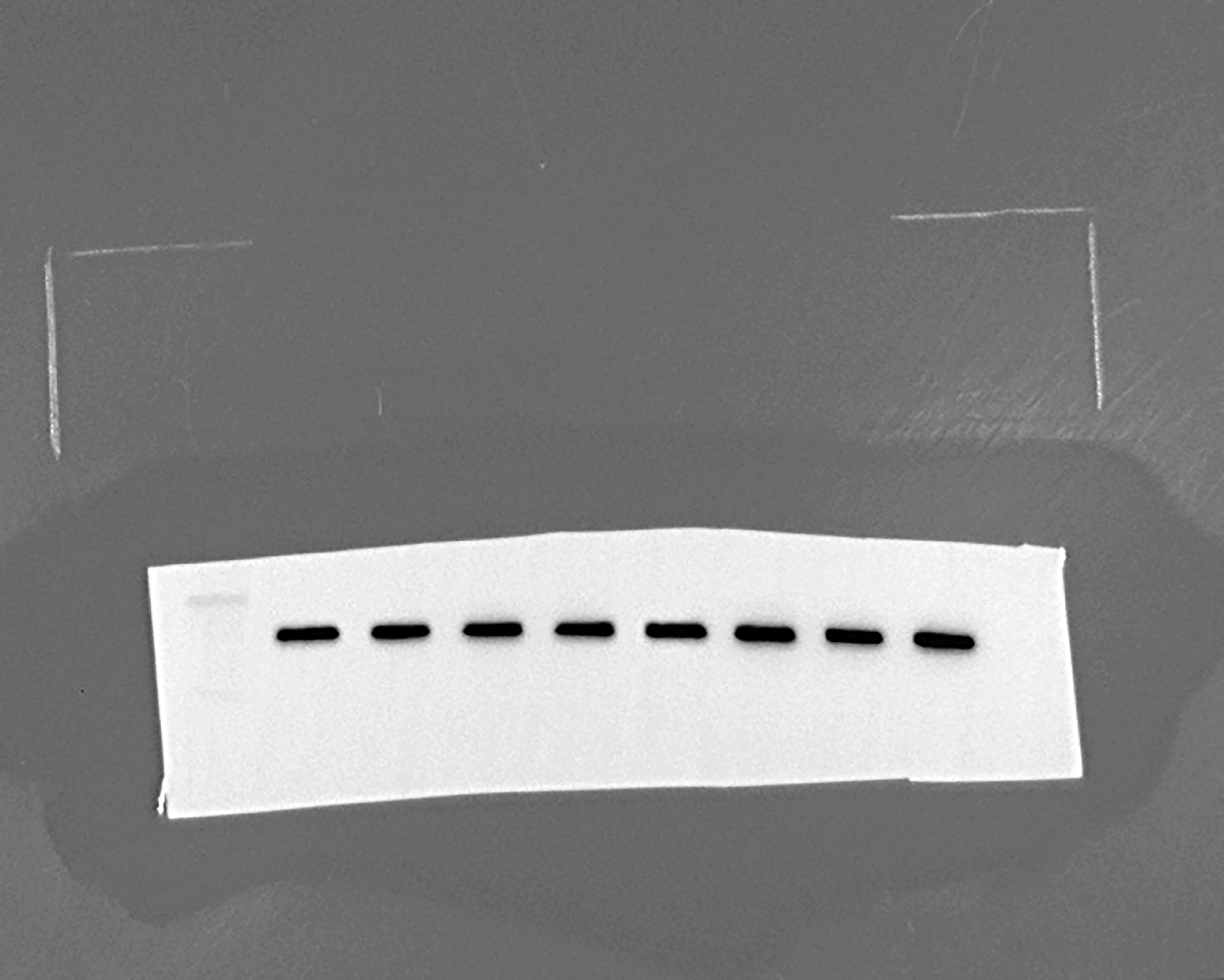

Supplement: Supplementary file 1 [file cancers-15-02065-s001.zip › File S1/a┬-Actin/a┬-Actin-1.tif]

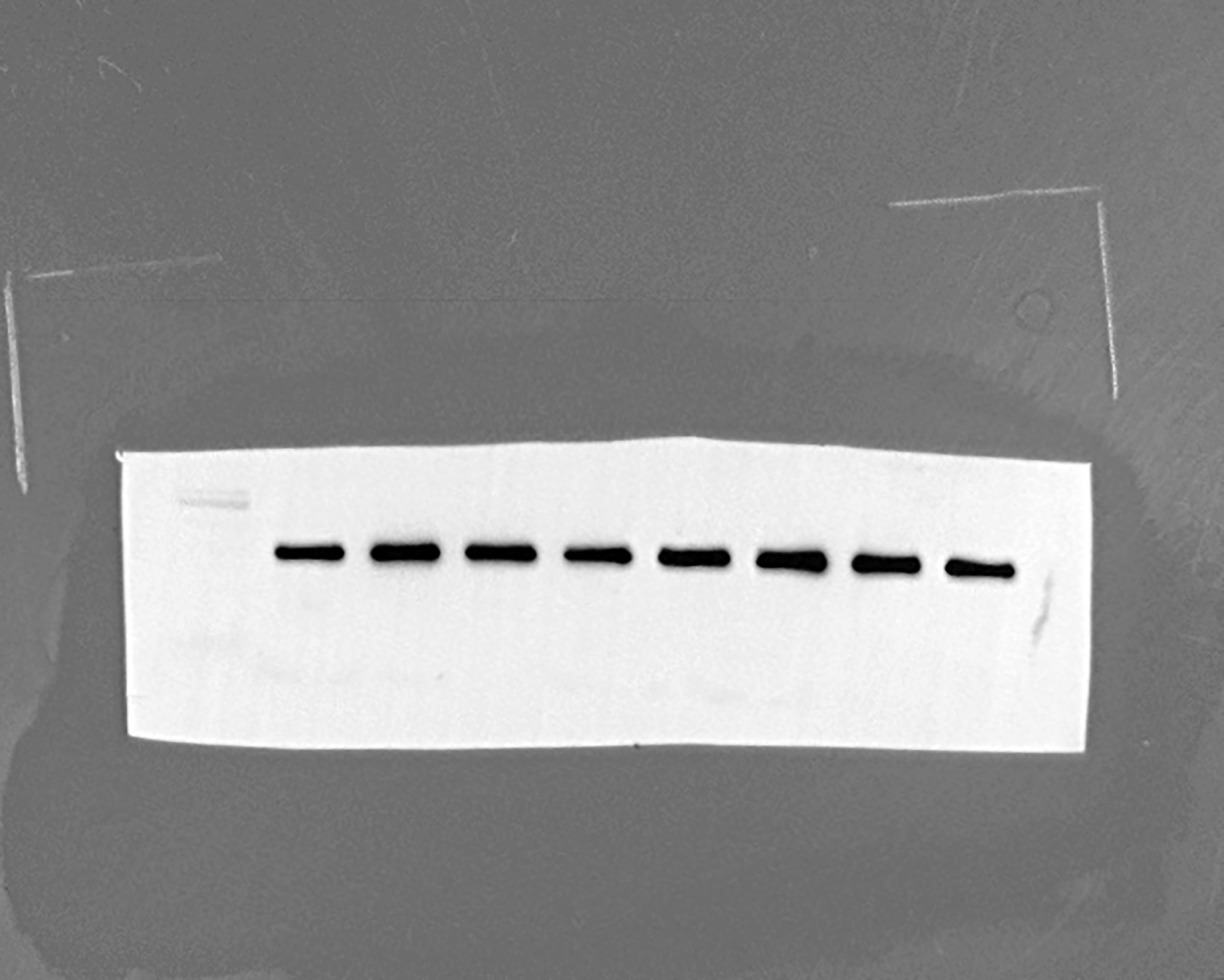

Supplement: Supplementary file 1 [file cancers-15-02065-s001.zip › File S1/a┬-Actin/a┬-Actin-2.tif]

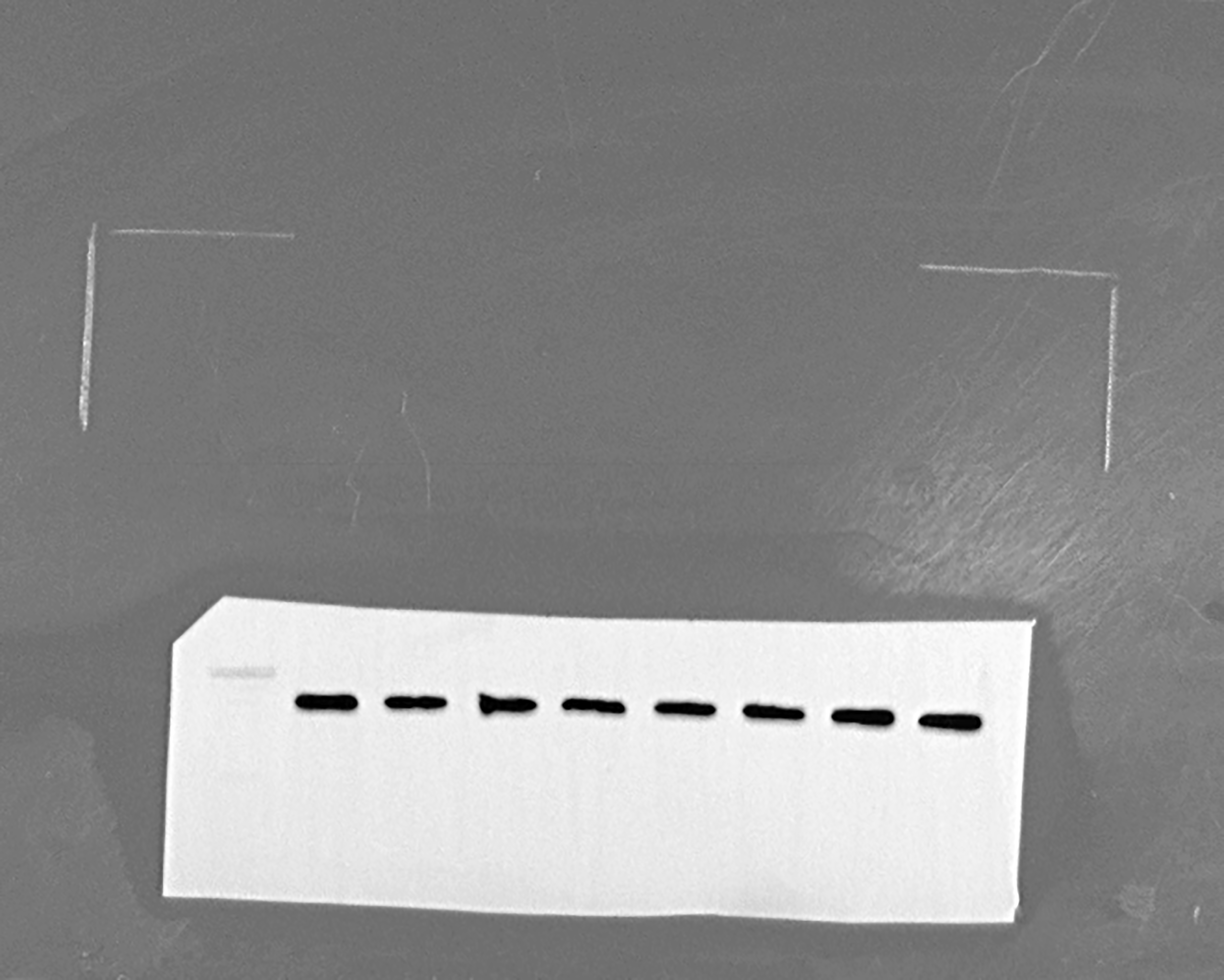

Supplement: Supplementary file 1 [file cancers-15-02065-s001.zip › File S1/a┬-Actin/a┬-Actin-3.tif]

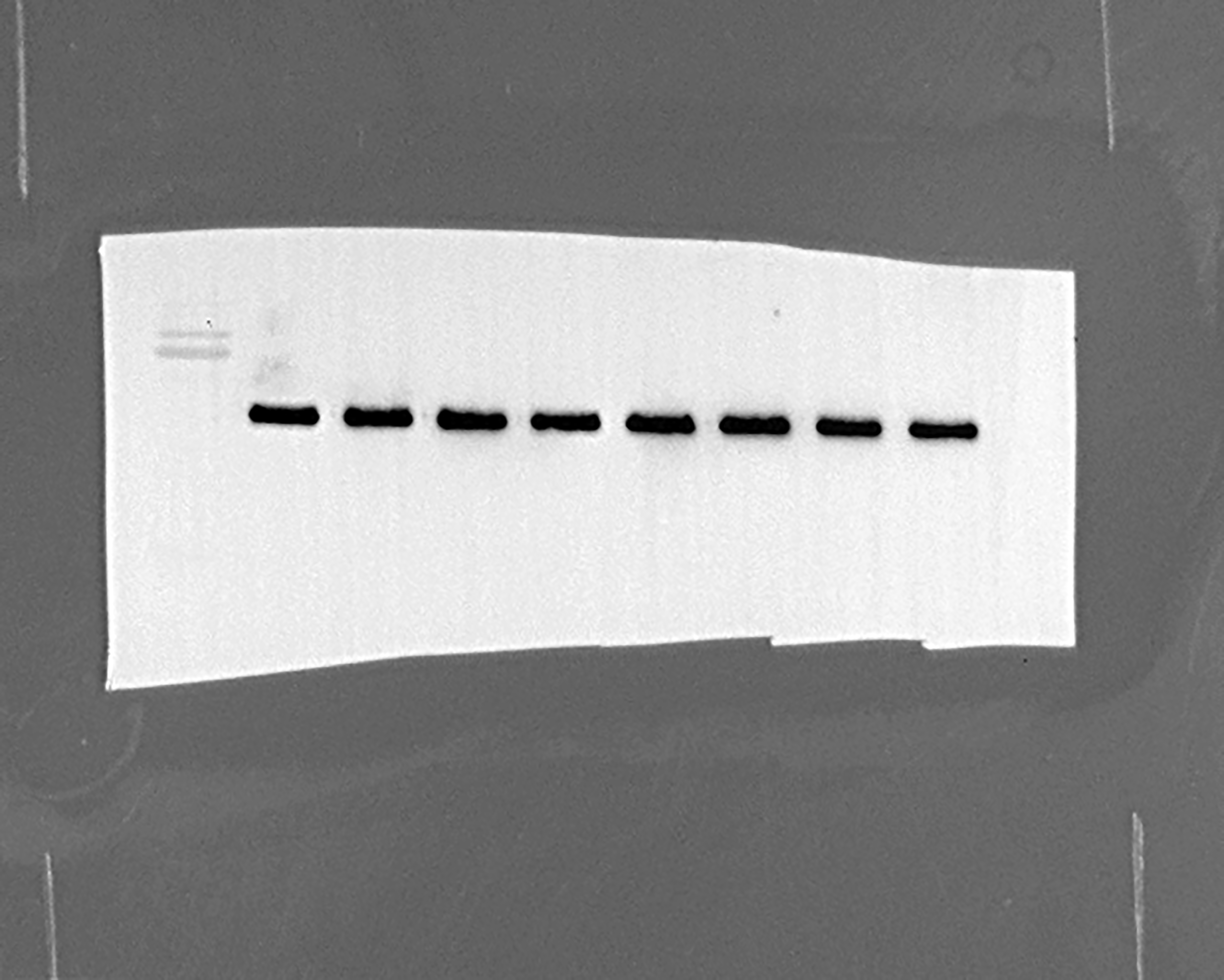

Supplement: Supplementary file 1 [file cancers-15-02065-s001.zip › File S1/a┬-Actin/a┬-Actin-4.tif]
